# Supplementary figures and images for: Age dependent collective trafficking of tissue resident T cells in zebrafish
Source: bioRxiv. 2026 Jul 15:2026.07.14.738523. Preprint. [Version 1] doi: 10.64898/2026.07.14.738523 (PMC13405546; doi:10.64898/2026.07.14.738523)

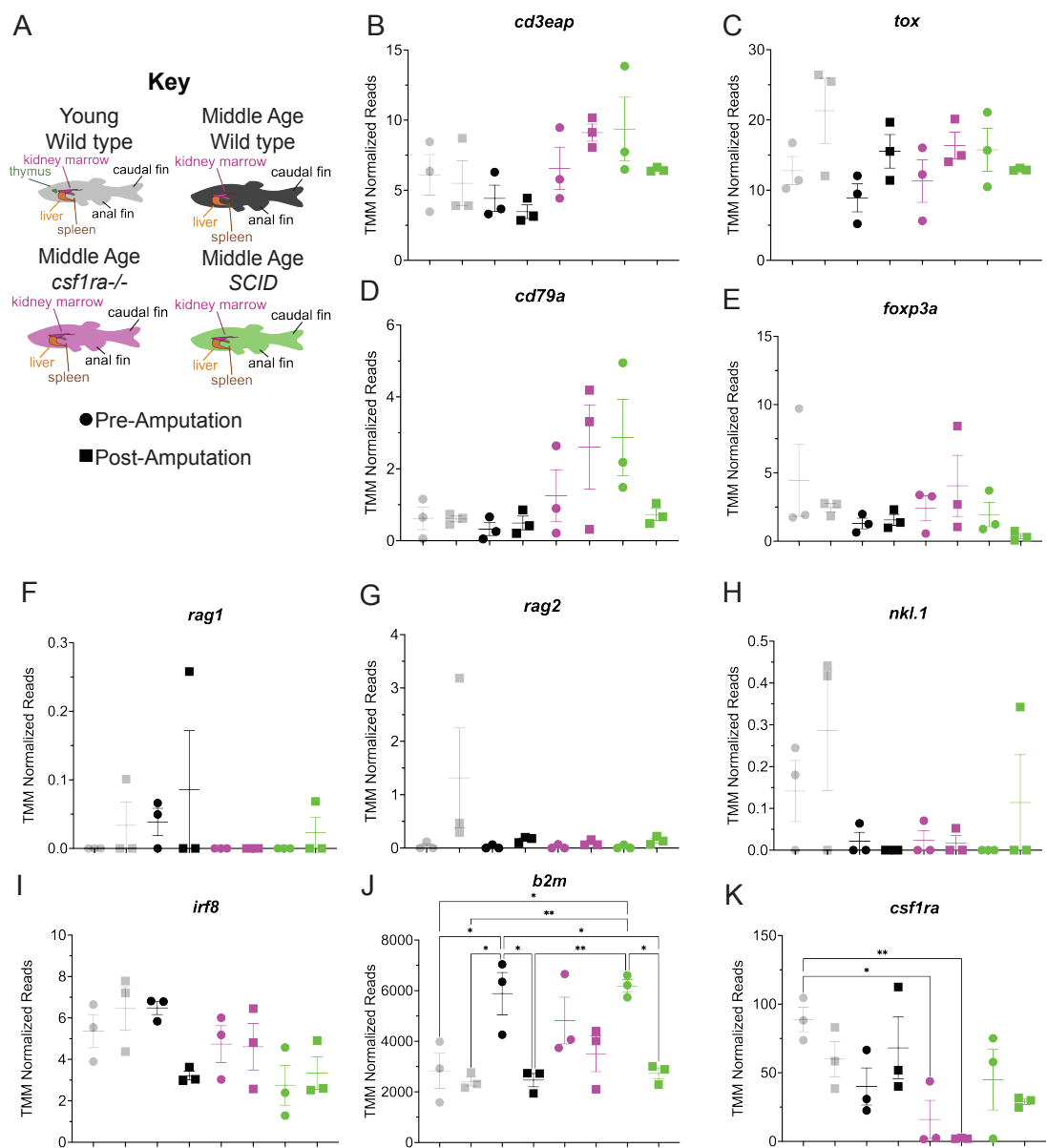

**Supplemental Figure 1**

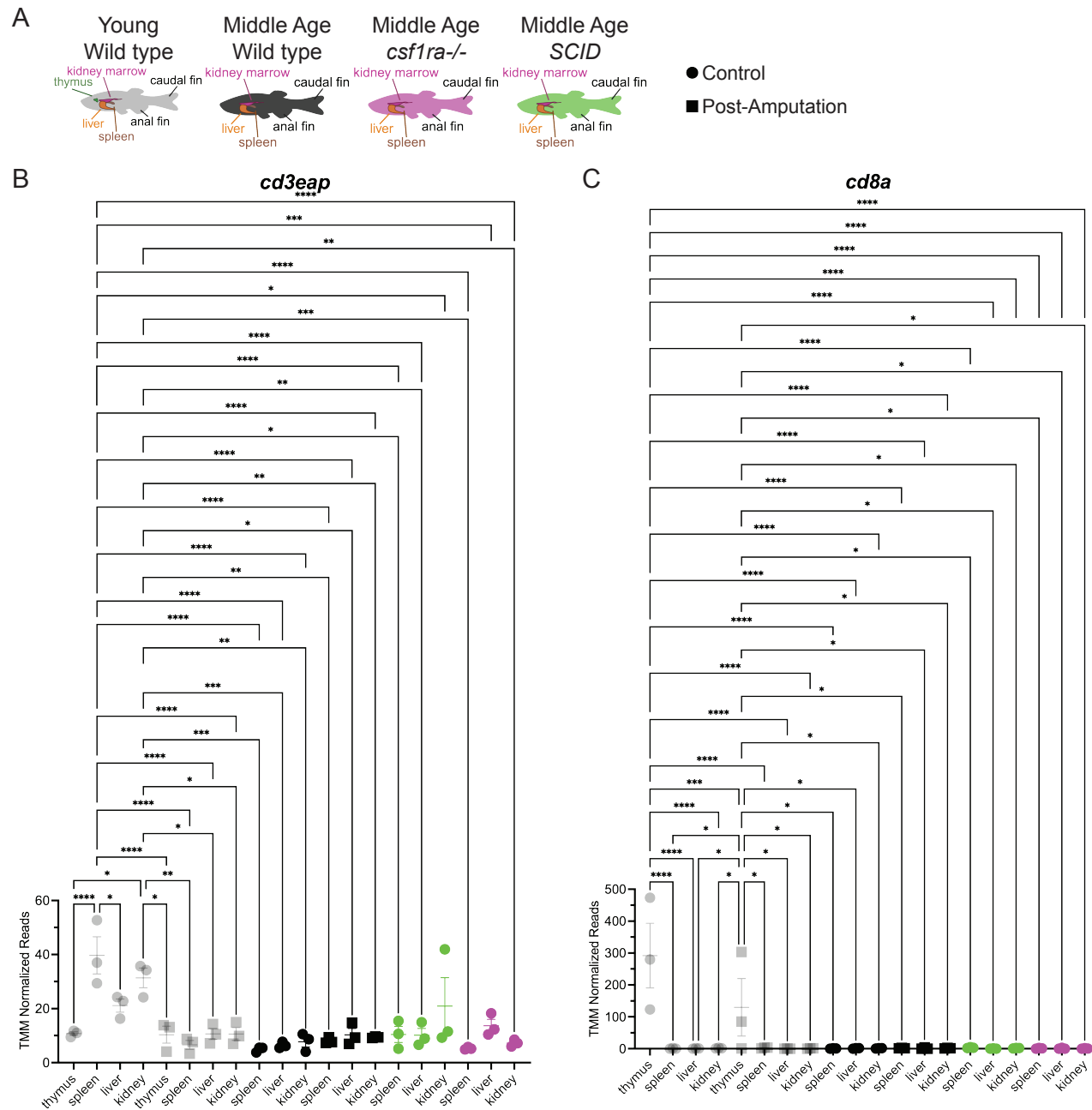

**Supplemental Figure 2**

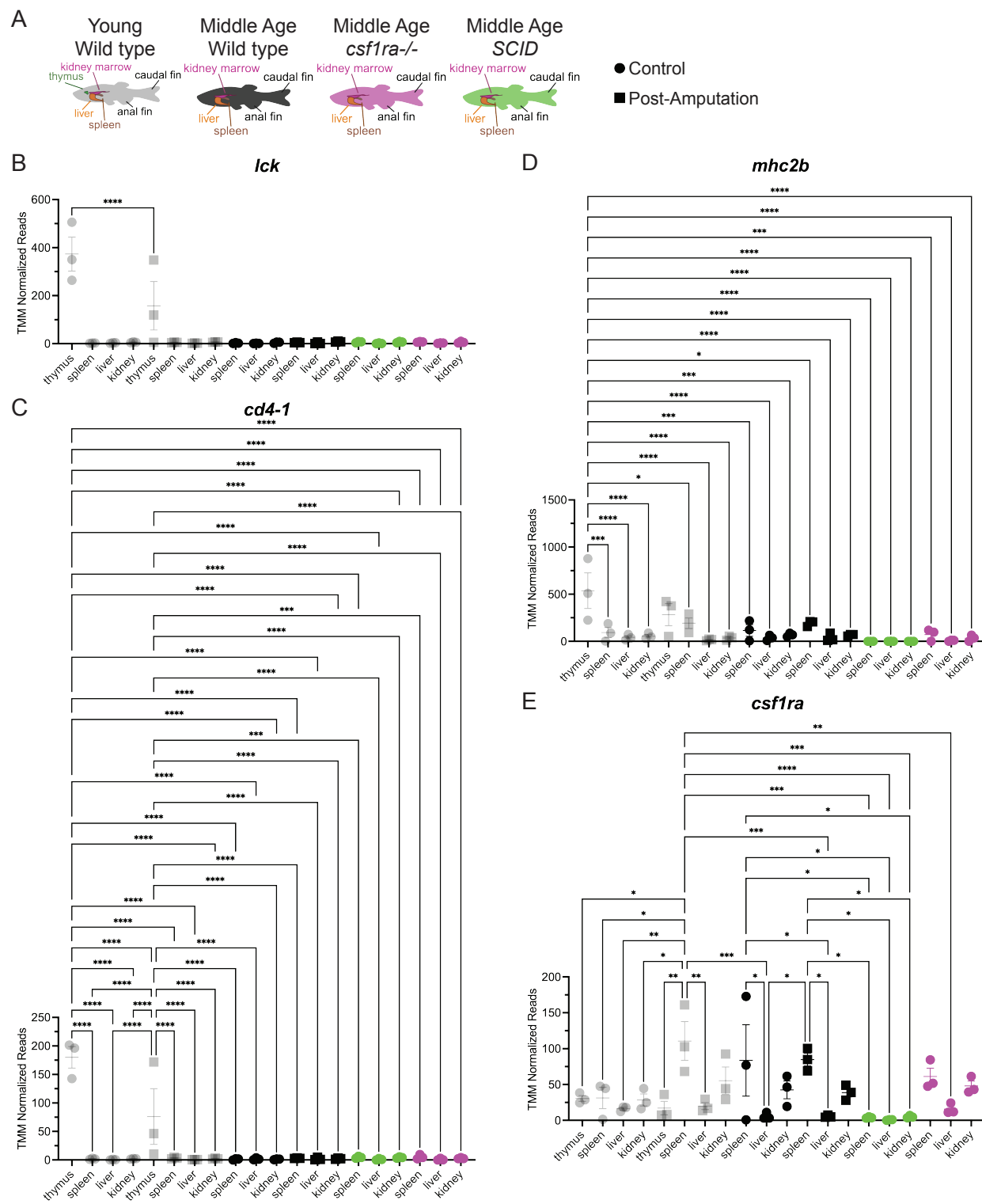

**Supplemental Figure 3**

Supplement: Supplement 2 [file media-2.pdf]
